# Impact of wall shear stress and ligand avidity on binding of anti-CD146-coated nanoparticles to murine tumor endothelium under flow

**DOI:** 10.18632/oncotarget.5662

**Published:** 2015-10-14

**Authors:** Stefan Thomann, Sunhwa Baek, Eduard Ryschich

**Affiliations:** ^1^ Department of Surgery, University of Heidelberg, Heidelberg, Germany; ^2^ Department of Pathology, University of Heidelberg, Heidelberg, Germany

**Keywords:** HCC, shear stress, nanoparticle, surface avidity, tumor endothelial marker

## Abstract

The endothelial phenotype of tumor blood vessels differs from the liver and forms an important base for endothelium-specific targeting by antibody-coated nanoparticles. Although differences of shear stress and ligand avidity can modulate the nanoparticle binding to endothelium, these mechanisms are still poorly studied. This study analyzed the binding of antibody-coated nanoparticles to tumor and liver endothelium under controlled flow conditions and verified this binding in tumor models *in vivo*. Binding of anti-CD146-coated nanoparticles, but not of antibody was significantly reduced under increased wall shear stress and the degree of nanoparticle binding correlated with the avidity of the coating. The intravascular wall shear stress favors nanoparticle binding at the site of higher avidity of endothelial epitope which additionally promotes the selectivity to tumor endothelium. After intravenous application *in vivo*, pegylated self-coated nanoparticles showed specific binding to tumor endothelium, whereas the nanoparticle binding to the liver endothelium was very low. This study provides a rationale that selective binding of mAb-coated nanoparticles to tumor endothelium is achieved by two factors: higher expression of endothelial epitope and higher nanoparticle shearing from liver endothelium. The combination of endothelial marker targeting and the use of shear stress-controlled nanoparticle capture can be used for selective intratumoral drug delivery.

## INTRODUCTION

Advances in nanotechnology enabled the development of nanoparticles with specific functional properties that address the shortcoming of traditional diagnostic and therapeutic agents. In contrast to single-molecule applications, one nanoparticle can carry a high amount of different imaging or therapeutic substances. Nanoparticle therapeutics such as immunoliposomal drugs and magnetic nanocarriers represent a rapid developing area in cancer therapy and some successful efforts in their clinical application have been achieved. For example, immunoliposomal doxorubicin formulation is clinically approved and used in treatment of different human cancer types [1, 2].

Superparamagnetic nanoparticles represent another class of nanoparticles that have been developed for diagnostic aims. These nanoparticles have been initially used only for magnetic imaging via passive tumor targeting, but recent advances have opened new opportunities for tumor-specific targeting and drug delivery [3]. The practicability of magnetic nanoparticles in purification and their potential implication in magnetic resonance imaging make these nanoparticles an intensively investigated subject. Current studies are mainly focused on the surface modification of nanoparticles to improve their bioavailability and tumor specificity. The additional coating of nanoparticles with tumor-specifc mAb increases specific drug delivery into the tumor [4].

So far, most of the nanoparticles have been developed to target molecules expressed on the tumor cell surface. For HCC, the potential value of EGFP-targeting immunoliposomes [5] and α-fetoprotein-targeting SNP [6] has been shown through the murine models. However, this type of strategy requires the transport of nanoparticles through the vascular wall and its subsequent diffusion in the extracellular matrix toward the tumor cells. In contrast to tumor cell-targeting strategies, vascular-targeting permits direct access to the tumor endothelial cell surface after systemic application of nanoparticles [7, 8].

**Figure 1:**
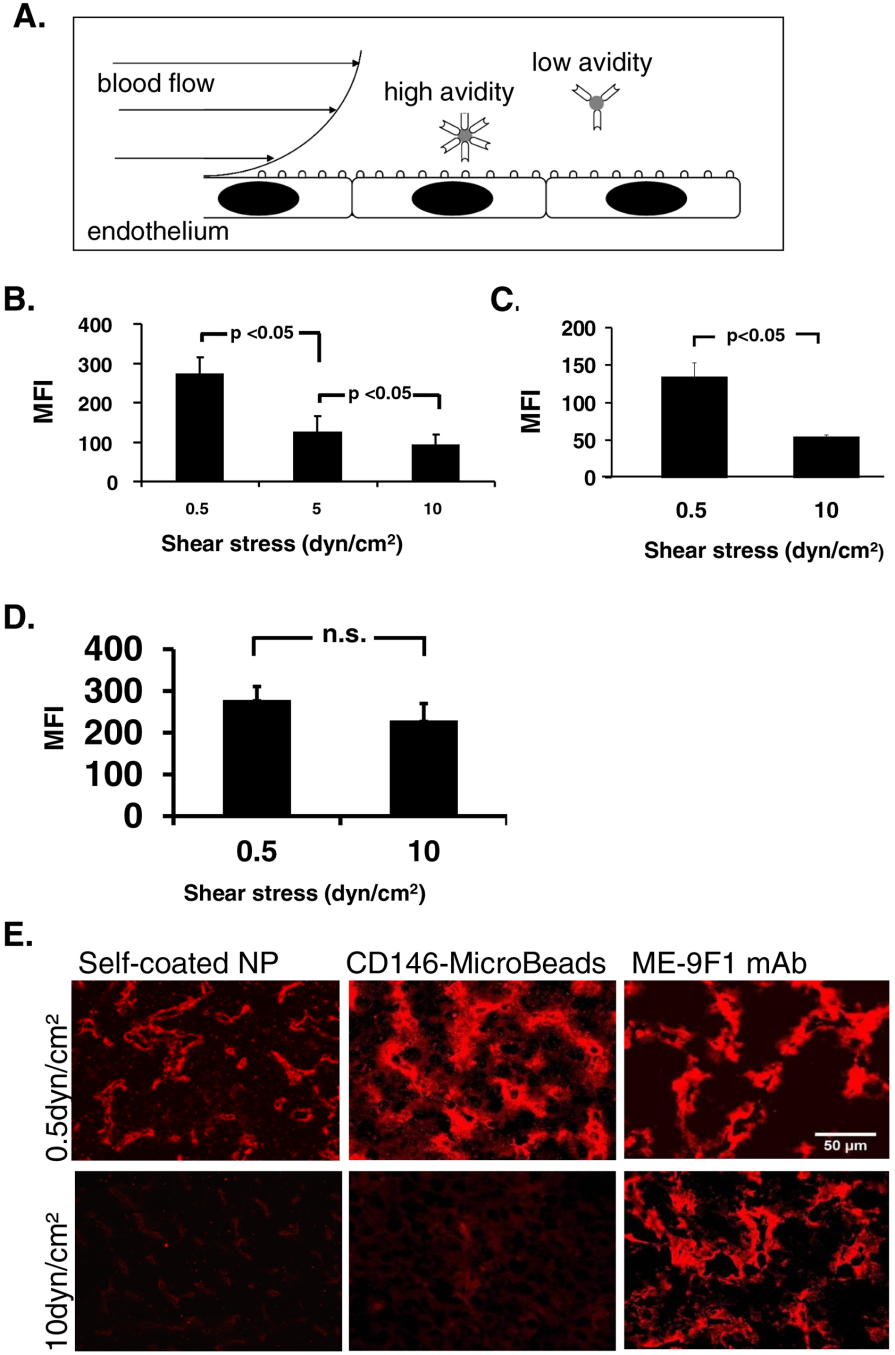
Influence of wall shear stress on nanoparticle binding to endothelium under flow **A.** Scheme of shear stress determinants in nanocarrier binding to endothelium. **B–D.** Binding of mAb-coated nanoparticles or mAb to endothelium on tissue slides was studied using a microfluidic chamber and image-based fluorimetry. Endothelial binding of self-coated nanoparticles (B) and CD146(LSEC)-Microbeads (C) decreased with increasing shear stress, whereas no significant difference of ME-9F1 mAb binding was found (D) **E.** Representative immunofluorescence imaging of nanoparticle and mAb binding to endothelium on microfluidic tissue slides. Fluorescence labeling of NP was performed by PE-conjugated secondary antibody. Scale bar (50 μm) as indicated.

Intravascular behaviour of coated nanoparticles differs sufficiently from single molecule binding because it additionally depends on hydrodynamic forces of microperfusion. In general, the binding of adhesive nanoparticles to endothelium follows the principle of avidity-dependent binding to endothelial cells as used by leukocytes adhering to endothelium (Fig. 1A) [7, 8]. According to this principle, leukocytes and endothelial cells express adhesion molecules under normal conditions, but the avidity of expression is low and thus, insufficient to support stable adhesion under shear stress in microvessels [9]. It has been previously demonstrated that the binding of anti-ICAM-1 mAb- [10] and glycocalicin-coated [7] nanocarriers to endothelium under flow is controlled by ligand avidity and shear stress. Previous studies of avidity- and shear stress-dependent binding of coated nanocarriers were mainly focused on normal endothelium. Although the shear stress and ligand avidity may significantly modulate the nanoparticle binding to tumor vasculature, this principle was only demonstrated using a computational model [11]. Experimental data of specific nanoparticle binding to tumor endothelium under different flow conditions and ligand avidities are still not available.

This study investigates the interrelationship of three individual factors: shear stress, avidity of endothelial epitope and nanoparticle coating, and their influence on nanoparticle binding to tumor endothelium *in vitro* and *in vivo*. CD146, an ubiquitous endothelial marker which is overexpressed in murine and human tumor tissue [12], was used as an endothelial ligand. A monoclonal antibody against CD146 (ME-9F1) was utilized as coating molecule for nanoparticle anchoring to its endothelial epitope.

## RESULTS

### The binding of mAb-coated nanoparticles, but not of mAb depends on wall shear stress

Specific binding of ME-9F1 mAb-coated nanoparticles and ME-9F1 mAb to endothelium on tissue slides was studied using a microfluidic chamber and image-based fluorimetry. It was found that the endothelial binding of self-coated nanoparticles with a coating avidity of 50% (see Table 1) decreased with increasing shear stress (Fig. 1B, *n* = 7). In particular, an increase of shear stress from 0.5 to 5 and from 5 to 10 dyn/cm^2^ significantly reduced the nanoparticle binding to tumor endothelium (*p* < 0.05, Fig. 1B).

**Table 1:** NP / mAb ratios for production of nanoparticles with different surface avidity

% of maximum avidity	Nanoparticles / ME-9F1 mAb ratio
100	1mg / 20 μg
50	1mg / 10 μg
25	1mg / 5 μg
12.5	1mg / 2.5 μg

Nanomag^®^-D-spio nanoparticles with a maleimide surface were conjugated with varying concentrations of thiolated ME-9F1 to produce antibody-coated nanoparticles with different coating concentrations. The nanoparticle coating ratio of 1 mg NP: 20 μg mAb was fluorimetrically defined as maximum avidity of 100%.

Next the capture of commercially CD146(LSEC)-MicroBeads was studied under 0.5 and 10 dyn/cm^2^ shear stress. These nanoparticles resembled the binding pattern of self-coatable nanoparticles and were well bound under low shear stress, and their binding under high shear stress was equally low (Fig. 1C, *n* = 3). In contrast to nanoparticles, ME-9F1 mAb binding did not show significant differences between shear stress rates of 0.5 and 10 dyn/cm^2^ (*n* = 2, Fig. 1D).

### Higher epitope avidity on tumor endothelium enhances nanoparticle binding compared to liver endothelial cells under laminar flow

**Figure 2:**
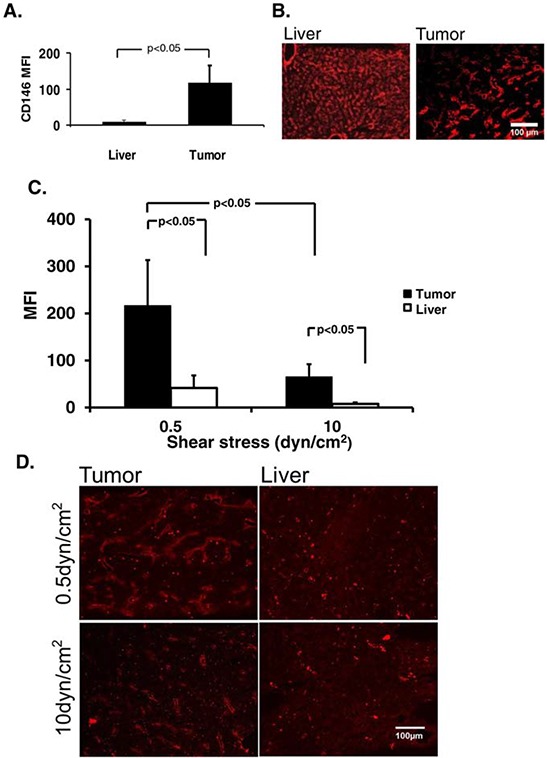
Avidity of endothelial epitope affects shear stress-dependent nanoparticle binding *in vitro* **A–B.** Expression of CD146 was significantly higher in tumor than in liver tissue. Quantitative analysis of CD146 mean fluorescence intensity (A) and representative immunofluorescence images (B). Scale bar (100 μm) as indicated. **C.** Differential nanoparticle binding to tumor and liver endothelium under flow. The nanoparticle binding to endothelium was significantly higher in tumor than in liver tissue both under high and low shear stress (*p* < 0.05), whereas the selectivity of nanoparticle binding (tumor/liver ratio) was increased at higher shear stress rates of 10 dyn/cm^2^. **D.** Representative immunofluorescence images of nanoparticle binding to tumor endothelium. Only autofluorescence (granules) was detected in liver tissue. Scale bar (100 μm) as indicated.

The increased expression of CD146 on tumor endothelium was verified using immunofluorescence and image-based fluorimetry. In accordance with previous studies, we found that CD146 was significantly higher expressed on tumor endothelium than on the liver (Fig. 2A–2B). Next, we compared the binding of ME-9F1-coated nanoparticles to liver and tumor endothelium under different shear stress rates. The nanoparticle binding to endothelium was significantly higher in tumor than in liver tissue both under high and low shear stress (*p* < 0.05, Fig. 2C–2D). Interestingly, the selectivity of nanoparticle binding was higher at 10 dyn/cm^2^ (tumor ÷ liver ratio = 8.5) than at 0.5 dyn/cm^2^ (tumor ÷ liver ratio = 5.2) (*p* < 0.05, Fig. 2C) (*n* = 5/group).

### Nanoparticle binding under flow correlates with coated mAb avidity

**Figure 3:**
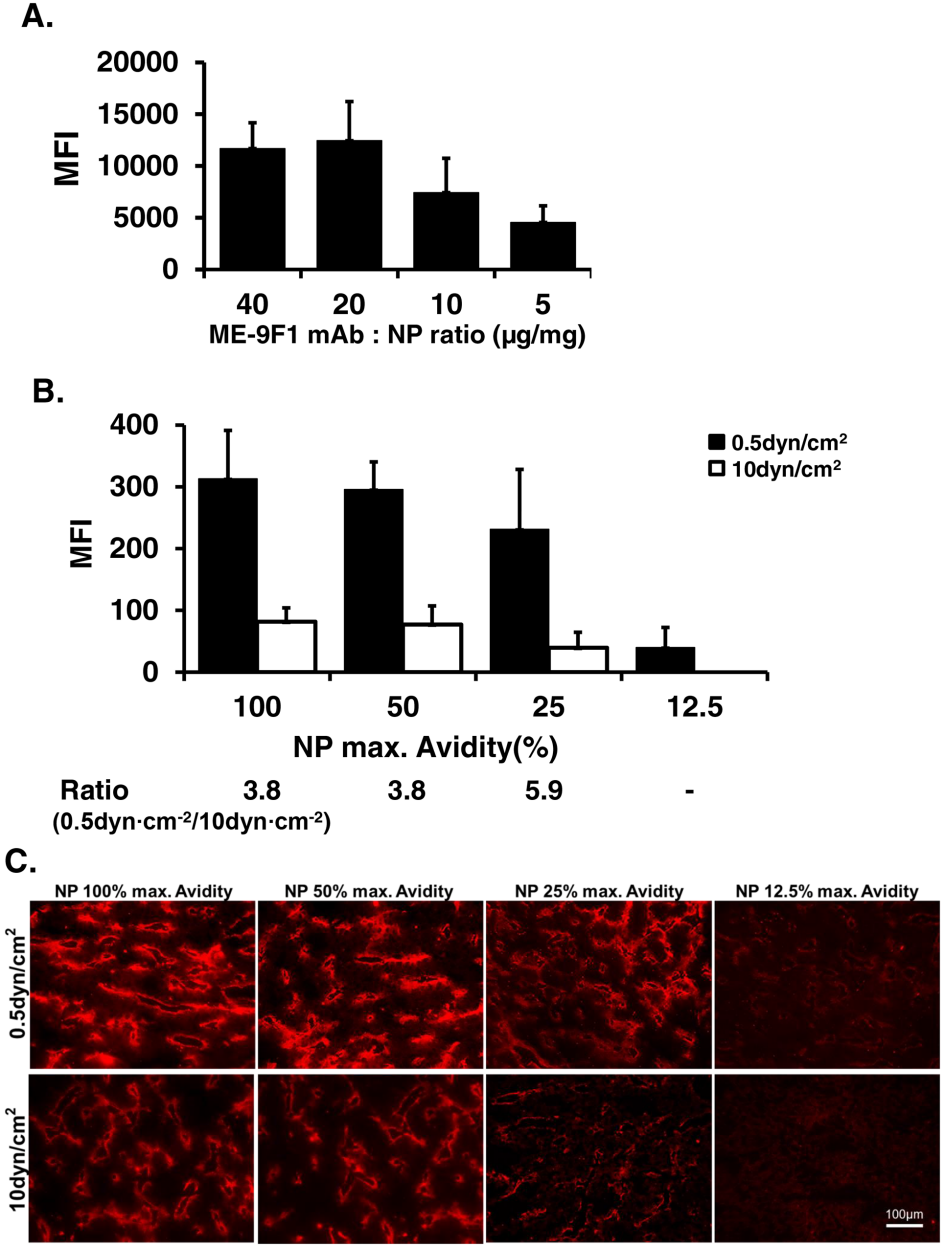
Nanoparticle coating avidity determines the degree of shear stress-dependent binding to endothelium under flow **A.** Nanoparticles with different coating avidity were produced. The amount of coated mAb was measured using fluorimetry. The coating efficacy increased with increasing amount of coating mAb from 10 to 20 μg/mg maleimide nanoparticle and achieved the saturation level at 20 μg per mg. **B–C.** The binding of these nanoparticles to endothelium under laminar flow at varying shear stress was studied. It was maximal at the highest coating density and at the lowest shear stress and continuously decreased with reduced coating density of mAb. Magnification bar (100 μm) as indicated.

To study the effect of coated mAb avidity on nanoparticle adhesion, nanoparticles with different coating densities were produced and analyzed. It was found that the coating efficacy enhanced as the amount of coated mAb was increased from 5 to 20 μg/mg maleimide nanoparticle and achieved the saturation level at 20 μg per mg (Fig. 3A).

Next, the binding of these nanoparticles to endothelium under laminar flow and varying shear stress was studied. Anti-CD146 nanoparticle binding was maximal at the highest coating density and at the lowest shear stress and continuously decreased with lower coating density of mAb (Fig. 3B–3C). The lowest coating density (12.5%) resulted only in detection of bound nanoparticles at 0.5 dyn/cm^2^, whereas no nanoparticle binding was detected at 10 dyn/cm^2^ (*n* = 3/group). The signal of bound NP with 25% avidity was almost 6 times higher at 0.5 dyn/cm^2^ than at 10 dyn/cm^2^ (Fig. 3B–3C).

### Tissue-specific biodistribution of ME-9F1 mAb-coated nanoparticles *in vivo*

**Figure 4:**
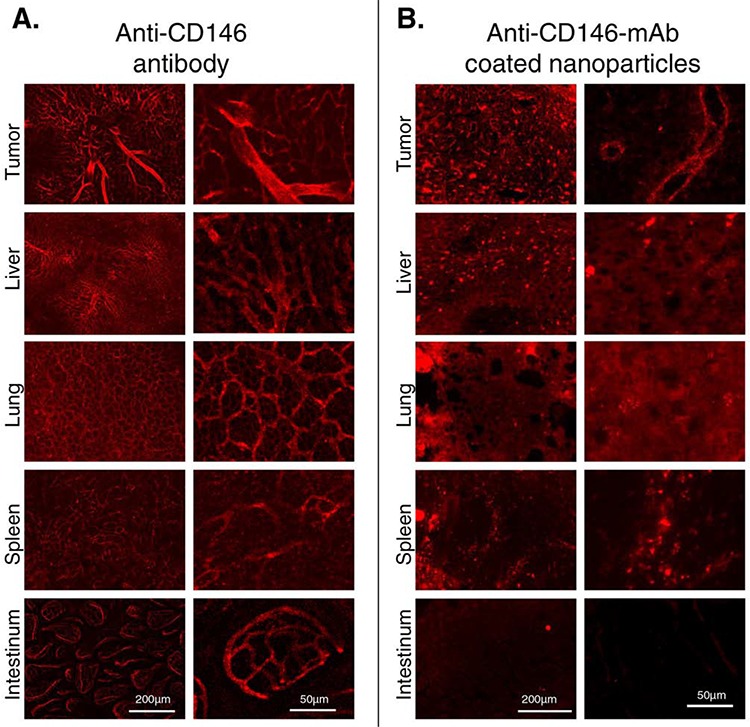
The binding of anti-CD146 mAb (A) and anti-CD146 coated nanoparticles (B) *in vivo* was studied using fluorescence microscopy Injection of mAb resulted in the staining of blood vessels in tumor, liver, lung, spleen and intestinal mucosa **(A)** Anti-CD146 coated nanoparticles were distinctly bound to tumor endothelium, whereas low binding in peritumoral liver was found **(B)** Images of two different magnifications are shown (see respective vertical rows) and magnification bars (50 or 200 μm) are indicated.

To examine the endothelial NP binding *in vivo* both NP and mAb were administered intravenously to tumor bearing AlbTag mice. Intravenous injection of ME-9F1 mAb resulted in the staining of blood vessels including tumor, liver, lung, intestinal mucosa and splenic blood vessels (Fig. 4A). Self-coated nanoparticles bound specifically to tumor endothelium, with low binding to liver vessels and partial endocytosis in peritumoral liver and spleen (Fig. 4B). The exact assessment of nanoparticle binding in lung tissue was not feasible because pulmonary capillaries were not clearly contrasted after nanoparticle injection. The nanoparticle binding in intestinal villi was almost absent (Fig. 4B).

## DISCUSSION

The present work is the first study which analyzed the relationship between nanoparticle binding to tumor and liver vascular endothelium and flow shear stress, as well as between avidity of nanoparticle coating and endothelial epitope avidity. The binding of nanoparticles was studied under static conditions as well as under laminar flow using shear stress of 0.5 dyn/cm^2^ and of 10 dyn/cm^2^. Shear stress of 10 dyn/cm^2^ approximately corresponds to physiological flow in the liver as shown in previous studies [13], whereas shear stress of 0.5 dyn/cm^2^ reflects very low flow conditions as a correlate of shear stress in dilated tumor vessels. CD146 was used as ligand on the endothelial surface for nanoparticle capture. It has been shown in previous work [13] and confirmed in this study that this endothelial marker is overexpressed on tumor endothelium in murine models of liver and pancreatic cancer [12]. It was found that the binding of coated nanoparticles and antibody was higher in tumor endothelium than in the liver both under near-static and high flow conditions. However, wall shear stress did not significantly influence antibody binding, whereas nanoparticle binding significantly varied in a shear stress-dependent manner. This binding pattern reflects a universal mechanism, as both self-coated pegylated and non-specialized (CD146(LSEC)-MicroBeads) nanoparticles showed identical binding properties under flow. Furthermore, the tumor:liver ratio of nanoparticle binding was substantially higher at shear stress of 10 dyn/cm^2^ (ratio = 8.5) than at 0.5 dyn/cm^2^ (ratio = 5.2). These results demonstrate two important findings. First, the nanoparticle binding to endothelium follows the principle of leukocyte adhesion under constant laminar flow. According to this principle, the avidity of interacting ligands on endothelium and on leukocytes control the number of adherent cells. This mechanism can be exemplified by the molecular interaction between LFA-1 on the circulating leukocyte and ICAM-1 on the endothelial surface [9, 14]. Pro-inflammatory stimulation causes the clustering of LFA-1 on cell surface which increases its avidity and promotes leukocyte binding to endothelium [9, 14].

Second, the intravascular wall shear stress impacts nanoparticle capture and favors selectivity of nanoparticle binding at the site of higher endothelial epitope avidity. Therefore, the overexpression of endothelial markers on tumor endothelium may additionally promote tumor-selective nanoparticle binding. The present study identifies endothelial surface marker overexpression as a relevant variable of nanoparticle binding by flow chamber experimentation and use of *in vivo* tumor models. We propose that for selective tumor endothelial targeting, the mAb avidity of the nanoparticle surface must be adjusted to the level by which physiological flow conditions selectively shear nanoparticles from normal endothelium and thereby promote preferential nanoparticle binding to the low shear stress environment of tumor microvessels.

Taken together, the selective binding of ME-9F1-coated nanoparticles to tumor endothelium is achieved by two factors: higher tumoral expression of endothelial epitopes and higher nanoparticle shearing from physiological liver endothelium. Furthermore, previous studies demonstrated that the averaged blood flow and wall shear stress are frequently decreased in tumors in comparison with normal tissue [15, 16]. Therefore, it can be concluded that the decreased shear stress in the morphologically abnormal tumor microvasculature can additionally contribute to the preferential enrichment of mAb-coated nanoparticles on tumor-associated endothelium.

For *in vivo* experiments, anti-CD146 mAb coated nanoparticles were systemically applied. These experiments confirmed the results of nanoparticle binding *in vitro*. They demonstrated that antibodies and nanoparticles have different distribution patterns: nanoparticles were preferentially bound to tumor endothelium, whereas antibody binding was homogeneously distributed in different organs according to the CD146 expression level.

Numerous previous studies described the general phenomenon of nanoparticle endocytosis and accumulation in hepatic and splenic tissue [17, 18]. It mainly occurs in LSEC, Kupffer cells and other macrophages [17] and substantially reduces the circulation time of systemically injected nanoparticles [19]. Many factors such as nanoparticle composition, size, geometry and surface charge influence nanoparticle endocytosis [3, 17, 18] certainly represents a problem for practical nanoparticle application *in vivo*. However, the improvement in production of more applicable nanoparticle types and in modification of the coating procedure itself can substantially prevent non-specific uptake as demonstrated by the use of alternative nanoparticle types in this study and in studies of other authors [19]. Nanoparticle surface pegylation is a common surface modfication for the improvement of the nanoparticle biocomatibility and circulation time [19]. Therefore, pegylated nanoparticles were used in the present study. As expected, systemic injection of pegylated NP led to reduced accumulation in RES of the liver and the spleen.

In the present study, iron-containg nanoparticles were used. These nanoparticles are very small (20 nm) and have superparamagnetic properties which simplify their preparation and handling e. g. repeated washing. Magnetic nanoparticles have been previously suggested for practical use in magnetic resonance imaging for tumor and vascular targeting [20]. Recently, a new persuasive method based on magnetic heating of iron-containing nanoparticles was introduced for treatment of human recurrent glioblastoma [21]. The results of the present study may provide important information on further development of magnetic nanoparticles which may help achieve higher intratumoral enrichment of coated nanoparticles. Production of nanoparticles with known coating avidity which is adapted to the tissue-specific shear stress may increase selectivity of local nanoparticle binding.

In summary, the selectivity of tumor-specific binding of mAb-coated nanoparticles depends on the epitope expression on tumor endothelium and on nanoparticle shearing from normal endothelium by shear stress. The targeting of endothelial markers such as CD146 and the use of shear stress-controlled nanoparticle capture can provide a useful tool for the selective drug delivery to tumor tissue.

## MATERIAL AND METHODS

### Mouse tumor models

Transgenic AlbTag mice were used at the stage of spontaneous HCC development between 10–12 weeks of age as previously described [22]. Tumor and normal tissue was dissected and shock-frozen in liquid nitrogen for histological preparation. All animal experiments were approved by the local committee of animal care (Regierungspräsidium Karlsruhe).

### Immunofluorescence and image-based fluorimetry

Endothelial CD146 expression was analyzed using 7 μm thick tissue sections of mouse liver and tumor tissue (*n* = 6) after staining with PE-conjugated anti-mouse CD146 mAb (ME-9F1, Biolegend, San Diego, USA). Mean fluorescence intensity (MFI) was measured as previously described [12] at three different areas per power field (three power fields per sample) containing at least five vessels and corrected by background fluorescence using Image J (NIH, Bethesda, USA).

### Nanoparticle coating procedure and fluorimetry avidity analysis

20 nm aminated pegylated magnetic nanoparticles (nanomag^®^-D-spio, Micromod, Rostock, Germany) were conjugated with ME-9F1 mAb using a three step process according to manufacturer's protocol. In brief, the coating protocol included activation of the maleimide nanoparticle surface, thiolation of the protein of interest with Traut's reagent and conjugation of the thiolated mAb with the maleimide activated nanoparticle. Antibody-coated nanoparticles were then magnetically separated in columns, washed and stored in PBS-EDTA buffer (0.01M PBS buffer, 1 mM EDTA, pH = 7.4).

For the generation of nanoparticles with different avidity of mAb coating, different quantities of thiolated mAb were processed with maleimide activated nanoparticles. The avidity of the nanoparticles was controlled by fluorescent detection of bound primary mAb to nanoparticles by PE-conjugated anti-rat IgG (clone Poly4054, Biolegend) and measured using fluorimetry (Fluostar Optima, BMG Labtech, Ortenberg, Germany).

### Microfluidic chamber

To analyze the nanoparticle binding under laminar flow, a microfluidic chamber was constructed using shock frozen fixed 7 μm thick tissue sections of liver or tumor tissue-coated coverslips (Knittelgläser Braunschweig, Germany). A sticky flow chamber (Ibidi Sticky-Slide I 0.4 Luer, Ibidi GmbH, Martinsried, Germany) was placed on top of the tissue specimen and additionally sealed using clips. A microfluidic chamber was perfused with 4 mg of self-coated or 80 μl of anti-CD146 mAb-coated nanoparticles (CD146(LSEC)-Microbeads, Miltenyi Biotec, Bergisch Gladbach, Germany, *n* = 3) dissolved in 8 ml of PBS-EDTA using a micropump system (Ibidi GmbH, Munich, Germany) under defined shear stress and laminar flow. To ensure a identical perfusion volume under different flow rates to each histological section, the microperfusion duration was modified and adapted (*n* = 7 for nanoparticles, *n* = 2 for mAb). Bound nanoparticles or mAb on the tissue section were stained using PE-conjugated anti-rat IgG (Biolegend) and quantified as described above.

### Nanoparticle injections in tumor-bearing mice and biodistribution analysis

To study nanoparticle binding *in vivo*, self-coated nanoparticles (6-7.5 mg/mouse, n = 4) were systemically injected in tumor-bearing mice. The tissue was dissected 30 min after injection, frozen and cut as described above. Nanoparticles were detected using immunofluorescence labeling with PE-conjugated anti-rat IgG mAb (Biolegend) and analyzed using fluorescence microscopy (Observer.Z1; Zeiss, Jena, Germany) or laser scanning confocal microscopy (Nikon A1R, Nikon Instruments, Düsseldorf, Germany). Antibody binding was studied using intravenous injection of PE-conjugated ME-9F1 mAb (500 ng/g BW, *n* = 3) and subsequent fluorescence microscopy of the whole-mount tissue as previously described [12].

## Abbreviations

HCC: hepatocellular carcinoma
MFI: mean fluorescence intensity
mAb: monoclonal antibody
BW: body weight
NP: nanoparticle
RES: reticuloendothelial system
LSEC: liver sinus endothelial cells
PE: Phycoerythrin
n. s.: not significant
